# Validation of Whole Slide Imaging in Pathology

**DOI:** 10.1155/2014/476854

**Published:** 2014-12-17

**Authors:** Liron Pantanowitz

**Affiliations:** Department of Pathology, University of Pittsburgh Medical Center, UPMC Cancer Pavilion, Suite 201, 5150 Centre Avenue, Pittsburgh, PA 15232, USA

## Abstract

Validation of whole slide imaging (WSI) is an important process for clinical practice. From a vendor perspective, validation seeks to achieve premarket approval from a regulatory agency in order for them to sell their WSI devices for an intended use (e.g., primary diagnosis). Academic validations are different, as these research studies typically result in publications reporting results of a self-defined, focused validation and their findings may not be generalizable. In general, published validation studies show good concordance for diagnoses made by WSI compared to glass slides. Clinical validation in the laboratory involves documenting the process and results of a validation study for an intended clinical use. This is typically conducted in accordance with available recommendations and/or guidelines. Validation studies comparing WSI (digital slides) to glass sides (traditional light microscopy) customarily measure concordance (agreement) between these two modalities. However, this outcome measurement can be influenced by several factors including validation study design, technology used, user training, observer variability, and case difficulty. Interobserver variability is likely to be greater than intraobserver variability, as not all pathologists may agree on certain diagnoses. Several guidelines have been developed that address WSI validation, which includes the College of American Pathologists (CAP) recommendations developed for validating WSI for diagnostic purposes. These CAP recommendations were subsequently adopted by the American Telemedicine Association (ATA) clinical guidelines for telepathology. Practical guidelines that assist pathology laboratories seeking to validate their own WSI systems for diagnostic work not only are timely but also help promote digital pathology adoption.

